# High-Dimensional Feature Selection for Automatic Classification of Coronary Stenosis Using an Evolutionary Algorithm

**DOI:** 10.3390/diagnostics14030268

**Published:** 2024-01-26

**Authors:** Miguel-Angel Gil-Rios, Ivan Cruz-Aceves, Arturo Hernandez-Aguirre, Ernesto Moya-Albor, Jorge Brieva, Martha-Alicia Hernandez-Gonzalez, Sergio-Eduardo Solorio-Meza

**Affiliations:** 1 Tecnologías de Información, Universidad Tecnológica de León, Blvd. Universidad Tecnológica 225, Col. San Carlos, León 37670, Mexico; mgil@utleon.edu.mx; 2 CONACYT, Centro de Investigación en Matemáticas (CIMAT), A.C., Jalisco S/N, Col. Valenciana, Guanajuato 36000, Mexico; 3 Departamento de Computación, Centro de Investigación en Matemáticas (CIMAT), A.C., Jalisco S/N, Col. Valenciana, Guanajuato 36000, Mexico; artha@cimat.mx; 4 Facultad de Ingeniería, Universidad Panamericana, Augusto Rodin 498, Ciudad de México 03920, Mexico; emoya@up.edu.mx (E.M.-A.); jbrieva@up.edu.mx (J.B.); 5 Unidad Médica de Alta Especialidad (UMAE), Hospital de Especialidades No. 1. Centro Médico Nacional del Bajio, IMSS, Blvd. Adolfo López Mateos esquina Paseo de los Insurgentes S/N, Col. Los Paraisos, León 37320, Mexico; martha.hernandez@imss.gob.mx; 6 División Ciencias de la Salud, Universidad Tecnológica de México, Campus León, Blvd. Juan Alonso de Torres 1041, Col. San José del Consuelo, León 37200, Mexico; sergio_solorio@my.unitec.edu.mx

**Keywords:** bank of features, coronary angiograms, evolutionary algorithm, feature selection, K-nearest neighbor, stenosis classification

## Abstract

In this paper, a novel strategy to perform high-dimensional feature selection using an evolutionary algorithm for the automatic classification of coronary stenosis is introduced. The method involves a feature extraction stage to form a bank of 473 features considering different types such as intensity, texture and shape. The feature selection task is carried out on a high-dimensional feature bank, where the search space is denoted by O$\left(2^{n}\right)$ and n=473. The proposed evolutionary search strategy was compared in terms of the Jaccard coefficient and accuracy classification with different state-of-the-art methods. The highest feature selection rate, along with the best classification performance, was obtained with a subset of four features, representing a 99% discrimination rate. In the last stage, the feature subset was used as input to train a support vector machine using an independent testing set. The classification of coronary stenosis cases involves a binary classification type by considering positive and negative classes. The highest classification performance was obtained with the four-feature subset in terms of accuracy (0.86) and Jaccard coefficient (0.75) metrics. In addition, a second dataset containing 2788 instances was formed from a public image database, obtaining an accuracy of 0.89 and a Jaccard Coefficient of 0.80. Finally, based on the performance achieved with the four-feature subset, they can be suitable for use in a clinical decision support system.

## 1. Introduction

**Figure 1:**
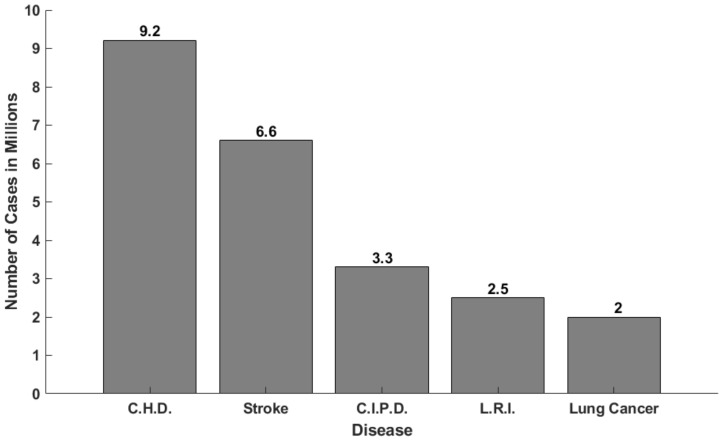
Comparison of five main diseases around the world: coronary heart disease (C.H.D.), stroke, chronic inflammatory pulmonary disease (C.I.P.D.), lower respiratory infections (L.R.I.) and lung cancer.

Coronary artery disease (CAD) stands as a leading cause of mortality in the majority of developed countries [1]. According to the British Heart Foundation (BHF) [2], coronary heart disease was the main cause of death in the year 2021 around the world. In Figure 1, a comparative chart of different death causes with data extracted from the BHF is illustrated.

According to Figure 1, coronary heart disease presents the highest rate of cases, with 9.2 million registered cases around the world.

**Figure 2:**
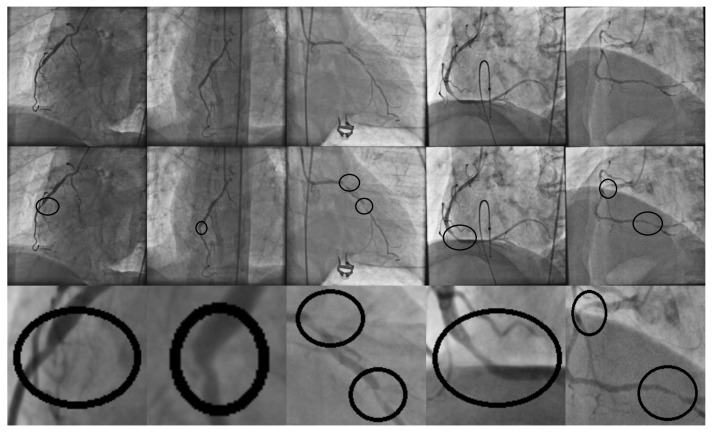
Coronary angiograms with their respective coronary stenosis regions labeled by the specialist.

In coronary artery disease, atherosclerosis leads to the development of coronary stenosis at various locations [3]. Nowadays, X-ray coronary angiograms are the gold standard for the detection of coronary stenosis in clinical practice. Consequently, a cardiology specialist must exhaustively examine the entire angiogram, and according to their expertise, all regions in which coronary stenosis cases can occur are labeled by hand. Figure 2 presents X-ray coronary angiogram samples with stenosis regions labeled by a specialist in cardiology.

The coronary stenosis problem has been studied in digital image processing, in which several challenging issues must be addressed, such as the presence of noise and weak contrast. In the literature, the method proposed by Saad [4] requires a previous vessel segmentation of a coronary angiogram to identify atherosclerosis using a vessel-width variation measure. A coronary stenosis measure grading method was proposed by Kishore and Jayanthi [5], using the pixel intensities of a previously enhanced image. Alternative methods, such as Brieva et al. [6], used a Hessian-based approach to extract different texture and shape vessel features to classify positive and negative stenosis cases.

Moreover, machine learning-based techniques have been used to address the stenosis classification problem. A naive Bayes classifier was used by Taki et al. [7] to classify calcified and non-calcified coronary artery plaques. The method of Cruz–Aceves et al. [8] uses a 3D intensity feature vector computed from the histogram of an image to detect a specific type of stenosis using a Bayesian-based classifier. Giannoglou et al. [9] proposed a fuzzy criterion for a feature selection process in atherosclerotic plaques. Chen et al. [10] use a 6D vector of shape features for the detection of coronary artery disease.

The main disadvantage of previous methods is the use of a fixed threshold measurement in order to classify coronary stenosis cases. Since image datasets of coronary angiograms have different size, contrast, and noise levels, the artery feature values present considerable variations. In consequence, the obtained results are highly dependent on the applied vessel enhancement method. In the proposed method, a number of spatial and frequency domain filters have been adopted for vessel enhancement in order to capture relevant information from different domains.

The method proposed by Antczak and Liberadzki [11], generates synthetic coronary stenosis and non-stenosis patches aiming to enhance the performance of a convolutional neural network (CNN) from scratch. Data augmentation techniques [12] are also used to generate a large number of instances that are used in the training and testing steps of a CNN. In addition, using the *explainable AI* concept, it is difficult to identify what features are really useful for correct classification and what they represent, which is the main drawback of the CNN [13]. Convolutional neural networks are still considered “black box” systems that do not offer any insight or explanations on how the decision is obtained, limiting the clinical applicability due to their lack of transparency and interpretability. Since the proposed method works with identifiable features, it is possible to know their relevance in the coronary stenosis classification by performance employing statistical analysis.

In the present paper, a novel strategy to perform high-dimensional feature selection using an evolutionary algorithm for the classification of coronary stenosis is proposed. The method involves a binary classification in order to identify positive and negative coronary stenosis cases. An automatic feature selection step is driven by a hybrid-evolutionary algorithm over a high-dimensional bank of features. The accuracy metric was used as a fitness function, while the discrimination rate was also maximized. Shape feature values such as vessel length, bifurcation points and others are dependent on a vessel enhancement technique. Therefore, different enhancement methods were applied to the original images. This strategy allows the formation of a feature bank of 473 different features. To obtain an optimal subset of features, an automatic feature selection stage is performed using a K-nearest neighbor classifier (KNN) to select relevant features. The classification of positive and negative stenosis cases is performed adequately in terms of the accuracy and Jaccard coefficients, which are useful to evaluate the rate of true-positive coronary stenosis cases and avoid the rate of true-negative cases. Since the problem involves a high-dimensional search space, which can be expressed as O$\left(2^{473}\right)$, a hybrid-evolutionary algorithm is appropriate for addressing the feature selection optimization problem. An evolutionary algorithm is a high-dimensional optimization technique for working in discrete and continuous domains using the Darwinian theory about the evolution of biological organisms throughout various generations. The hybrid-evolutionary strategy achieved a discrimination rate of 0.99, obtaining a subset of four features from the extracted bank of 473. In the experiments, a database containing 608 images was used for training of the proposed method (508 for training and 100 for testing). In addition, a second public domain database [11], of 2788 coronary patch images was used for testing.

The rest of this paper is structured as follows. The background methods of vessel enhancement methods, along with intensity, texture, and shape features, are presented in Section 2. In Section 3, the bank of 473 features, the hybrid-evolutionary algorithm, and the performance metrics of the proposed method are introduced. Experimental results are described in Section 4, and conclusions are presented in Section 5.

## 2. Materials and Methods

### 2.1. Experiment Materials

For the experiments, two distinct banks of images were used. The first image database was formed from a bank of images provided by the Mexican Institute for Social Healthcare and authorized by an institutional review board only for research purposes under the reference R-2019-1001-078. The bank contains 180 digital images of coronary angiograms, which are 512×512 pixels and have a grayscale color scheme. It also included the corresponding ground-truth images with the coronary stenosis cases labeled by a cardiology specialist. From the provided image bank, 304 patches of size 64×64 pixels containing coronary stenosis cases were extracted. In addition, 304 additional patches with non-stenosis cases were extracted in order to form a balanced database. A total set of 608 image patches were extracted to form the first database. The size of the patches corresponds to the area enclosing the stenosis cases and was labeled and validated by the specialist.

In order to assess the obtained results, a second image database was formed from the Antczak [11] image database, which is in the public domain. Each image corresponds to a coronary patch of size 32×32 pixels in a grayscale color scheme. The original bank is formed by 122 natural coronary patches containing a stenosis case. In addition, it contains 1394 natural coronary patches with non-coronary stenosis cases. Since the proportion of positive and negative stenosis cases is unbalanced, 1272 additional synthetic patches with a coronary stenosis case were added, which were taken from the same image bank. Consequently, the second image database consists of 2788 images with a balance of positive and negative coronary stenosis cases.

### 2.2. Feature Extraction

The description and measurement of objects of interest, properties of an image, or a specific region is commonly known as feature extraction [14]. It is possible to extract distinct feature types, as described in the literature [15,16]. According to their nature, features can be classified into texture, intensity, and shape.

#### 2.2.1. Intensity Features

Intensity features are relevant in digital image processing because they are related to the corresponding value for each pixel in the image. Here, the five minimum, maximum, median, average, and standard deviation statistical measures of the pixel intensity have been extracted.

#### 2.2.2. Texture Features

Texture features are relevant in different cardiovascular problems [17,18]. One of the most used approaches for the extraction of texture-related features over images is the gray-level co-occurrence matrix (GLCM) [19]. The GLCM computes the frequency of variation between the intensity levels of a pixel. It is expressed as a matrix whose rows and columns correspond to the entire image’s pixel intensities. The frequencies of intensity variations are computed in a specific spatial relationship denoted by (Δx,Δy) between two different pixels with intensity levels *i* and *j* as follows:

(1) $$C_{\Delta x,\Delta y}\left(i,j\right)=\sum_{x=1}^{n}\sum_{y=1}^{m}\left\{\begin{array}{c} 1,\mathrm{if}\;I(x,y)=i\;\mathrm{and}\;I(x+\Delta x,y+\Delta y)=j\\ 0,\mathrm{otherwise} \end{array}\right.,$$

where $C_{\Delta x,\Delta y}\left(i,j\right)$ is the frequency in which two pixels with intensities *i* and *j* at a specific offset (Δx,Δy) occur, and *n* and *m* represent the height and width of the image.

Additionally, it is also possible to extract texture features from the output of alternative representation methods, such as the Radon transform [20]. The Radon transform is the projection of the image intensity along with a radial line oriented at some specific angle, which can be computed as follows:

(2) $$R\left(\rho ,\theta \right)=\int_{-\infty }^{\infty }\int_{-\infty }^{\infty }f\left(x,y\right)\delta \left(\rho -x\;\cos\;\theta -y\;\sin\;\theta \right)dxdy,$$

where R(ρ,θ) is the Radon Transform of a function f(x,y) at an angle θ, δ(r) is the Dirac delta function, and δ(ρ−xcosθ−ysinθ) forces the integration of f(x,y) along the line ρ−xcosθ−ysinθ=0.

#### 2.2.3. Shape Features

Shape-based features enable the extraction of quantifiable information concerning various aspects related to the artery shape; for example, a segment length, the tortuosity level present in a determined arterial section, or the number of bifurcations present on it and the vessel width. Nevertheless, to extract shape-based features, it is necessary to perform a previous vessel enhancement process over the original image in which useless information such as noise and background are identified. Consequently, the shape measure values are highly dependent on the applied filtering method. The use of the Hessian matrix and eigenvalues methodology [21] has proved to be adequate for vessel enhancement. However, an automatic thresholding strategy such as the Otsu method [22] is necessary to separate vessel and non-vessel pixels.

The vessel skeleton has been used to extract vessel-shape-related information. To obtain the corresponding vessel skeleton from a binary segmented image, the medial-axis transform method has been commonly applied [23].

### 2.3. Vessel Enhancement Methods

Vessel enhancement methods are useful for discriminating irrelevant non-vessel information on coronary angiograms. In the literature, spatial and frequency domain filters have been applied to the vessel enhancement problem, achieving suitable results. In the experiments, eight state-of-the-art vessel enhancement methods have been adopted, which are mentioned below.

1. Spatial domain filters
  (a) Hessian-based methods.
    - Vesselness measure [24]. The Frangi method computes a vesselness measure using the eigenvalues of a Hessian matrix.
    - Hessian matrix and clustering [25]. The Hessian matrix is also used by Salem et al. for vessel enhancement by computing the largest eigenvalue and vessel orientation over all scales.
  (b) Morphological top-hat filter [26]. In mathematical morphology, the top-hat filter is useful to enhance images with non-uniform illumination. Because of this property, the top-hat operator has been used to enhance vessel-like structures [27,28].
  (c) Multi-scale line detection [29]. An alternative approach that has been used for artery enhancement is the linear matched filter. This method works under the assumption that blood vessels can be modeled by linear segments that share the same orientation and length.
  (d) Gaussian matched filter
    - Single-scale Gaussian filter (GMF). In this approach, a gray-scale template is formed from a Gaussian distribution, which is convolved with the input image.
    - Multi-scale Gaussian filter. The main limitation of GMF is the use of a fixed vessel diameter represented by the σ parameter in which non-corresponding vessel diameters will be distinguished. In order to overcome this disadvantage, a multi-scale Gaussian matched filter was proposed by Cruz–Aceves et al. [30] considering different vessel width scales.
2. Frequency domain filters
  (a) Gabor filter
    - Single-scale Gabor filter [31]. The Gabor filter is a Gaussian curve modulated by a sinusoidal function, which is useful for the detection of directional features. In addition, Rangayyan et al. [32] simplified the matching template equation so it is governed by only two parameters.
    - Multi-scale Gabor filter [33]. Similar to the GMF, the use of a fixed vessel diameter represented by the τ parameter will only detect the main artery tree and, as a consequence, discriminate vessels with diameters lower than τ. In order to overcome this disadvantage, Rangayyan et al. proposed a multi-scale Gabor filter for retinal vessels.

### 2.4. Metaheuristics

The classification techniques learn and predict by classifying instances defined by their features. Therefore, the classification accuracy performance is highly dependent on the used feature set since not all of the used features could be relevant for the classification process. In this context, a feature selection task is necessary after the feature extraction stage is performed. However, the feature selection task is turned into a high-dimensional complexity problem when the number of involved features is elevated because the number of different combinations that are required to find the most suitable feature subset is denoted by $2^{n}$, where *n* is the number of involved features. Consequently, the use of high-dimensional optimization algorithms is appropriate to address the feature selection problem.

#### 2.4.1. Simulated Annealing

Simulated annealing (SA) is a metaheuristic that was abstracted from an industrial process. In the annealing process, the material is exposed to a certain high temperature, and after, a controlled cooling process is performed. Simultaneously, care must be taken in order to preserve certain molecular alignments in the material to ensure their quality. This process was adapted as a computational search technique by Kirpatrick et al. [34] to solve combinatorial and continuous optimization problems. The algorithm is governed by the $T_{min}$, $T_{max}$ and $T_{step}$ parameters, which refer to the minimum, maximum, and changing-step temperatures, respectively. At each iteration step, a new solution is generated based on a computed probability that involves the current temperature and the decreasing parameter ΔE, which represents the objective function response. The probability is calculated from the Boltzmann distribution as follows:

(3) $$P\left(\Delta E,T\right)=\frac{f\left(s^{\prime }\right)-f\left(s\right)}{T},$$

where P(ΔE,T) is the probability computed from the Boltzmann distribution, and $f(s^{\prime })$ and f(s) denote the objective function value obtained with the current and the previous SA solution, respectively.

#### 2.4.2. Boltzmann Univariate Marginal Distribution Algorithm (BUMDA)

BUMDA [35] is a population-based method that uses the estimation of distribution to generate new individuals. The main idea of BUMDA is the use of a distribution probability computed from the best solutions of the current generation in order to generate the new one [36]. The Boltzmann probability distribution used by BUMDA is calculated as follows:

(4) $$\mu =\sum_{j}W\left(X_{j}\right)x_{j},\;\mathrm{where}\;W\left(X_{j}\right)=\frac{g(X_{j})}{\sum_{X_{j}}g\left(X_{j}\right)},$$

(5) $$\nu =\sum_{j}W^{\prime }\left(X_{j}\right){\left(X_{j}-\mu \right)}^{2},\;\mathrm{where}\;W^{\prime }\left(X_{j}\right)=\frac{g(X_{j})}{\sum_{X_{j}}g\left(X_{j}\right)+1},$$

where μ is the objective function average, ν is the objective function variance that was obtained from the population. $g(X_{j})$ corresponds to the value of the objective function obtained by the individual $j^{th}$, which is an individual of the population *X*. Consequently, in order to generate the next population, a fraction of the current one that contains the best individuals is used to produce the new generation, as follows (npop is the population size):

(6) $$\theta^{t+1}=\left\{\begin{array}{cc} f(x_{\mathrm{npop}}) & \mathrm{if}\;t=1,\\ f(x_{\frac{\mathrm{npop}}{2}}) & \mathrm{if}\;f\left(x_{\frac{\mathrm{npop}}{2}}\right)>=\theta^{t},\\ f(x_{i}) & \mathrm{when}\;f\left(x_{i}\right)>=\theta^{t}{|}_{i=\frac{\mathrm{npop}}{2}+1}^{\mathrm{npop}}, \end{array}\right.$$

### 2.5. Machine Learning-Based Classifiers

Classifiers are useful for deciding if a specific instance belongs to part of one class or another. For the coronary stenosis classification problems, they are useful for determining if an image or a region over it corresponds to a positive stenosis case or a negative one.

#### 2.5.1. K-Nearest Neighbor

K-nearest neighbor (KNN) represents a fast classification method that was first proposed by Evelyn Fix and Joseph Hodges in 1952 [37]. Later, in 1967, Thomas Cover and Peter E. Hart expanded the initial proposal by introducing the concept of *nearest neighbor* [38]. The KNN is governed only by the *k* parameter, which is a positive integer and indicates the number of associated nearest neighbors that a new instance will have in order to measure its probability of membership to different classes. The KNN inputs are labeled vectors in a multidimensional feature space. In the first stage, the training of the KNN model consists only of the storage of the feature vectors and their corresponding class. In the second stage, the KNN classifies new instances by measuring their frequency among the *k* nearest instances to determine the label of the new instance. In addition, the “nearest” term is associated with the similarity concept in which measurement is commonly based on a distance metric (commonly, Euclidean distance).

#### 2.5.2. Support Vector Machine

Originally conceived as a linear separator for binary classification in supervised learning, the Support Vector Machine (SVM) faces challenges when dealing with instances characterized by significant data overlaps, making linear separability unattainable [39]. To address this issue, SVM has the capability of projecting instances from their original representation space into higher-dimensional orders, enabling successful classification [40]. To execute these projections, SVM leverages instances situated on both sides of the separation boundary, whether it be a line, plane, or hyperplane. The SVM is then formulated as follows [41]:

(7) $$f\left(x\right)=W^{T}\phi \left(X\right)+b,$$

where *W* is the weight vector and normal to the hyperplane, ϕ is the projection function or kernel, *b* is the bias or threshold, and *X* is the data point to be classified.

## 3. Proposed Method

**Figure 3:**
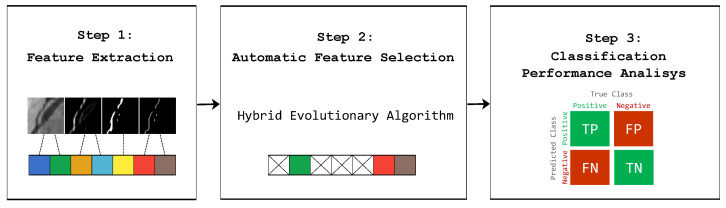
Proposed feature extraction, selection, and classification methods to classify coronary stenosis.

The proposed strategy consists of three stages. The first stage corresponds to the feature extraction in order to form a bank of 473 features involving intensity, shape, and texture types. For shape features, 8 vessel enhancement methods from the state-of-the-art were used. In the second stage, a subset of features is selected (feature selection) by using a hybrid-evolutionary algorithm in order to maximize the classification accuracy in training data while minimizing the number of features. In the final stage, the selected subset is tested for the classification of coronary stenosis using an independent test set of angiograms. In Figure 3, the steps of the proposed strategy are illustrated.

The bank of 473 features is formed as follows. Intensity-based features, such as minimum, maximum, median, mean, and standard deviation of the pixel intensities, are present in the original image. In addition, these features were also extracted from the responses of the different enhancement methods. In texture features, the Haralik [42] methodology was applied in order to obtain 14 distinct texture-related features. Consequently, 50 different shape features were extracted, including those used by Welikala [43]. Since shape feature values depend on the previous image enhancement process used, different methods were applied, such as those by Frangi et al. and Salem et al., as well as single and multi-scale Gaussian matched filtering, single and multi-scale Gabor filtering, linear multi-scale, and a multi-scale top-hat operator. All extracted features are described below.

**Intensity-based Features**

The intensity features correspond to the statistical measures of standard deviation, minimum, maximum, average, and median of the pixel intensities. Those features were computed from the original image and from the filter response of the 8 enhancement methods described previously. Consequently, 45 intensity-based features were extracted.

**Texture Features**

From the original image, the set of 14 texture features proposed by Haralik [42] were computed. In addition, this set of features was also applied to the Radon transform response. The total number of texture features is 28.

**Shape Features**

For shape features, 14 of them were extracted as described in the Welikala methodology [43], as follows:

1. The total number of vessel pixels.
2. The total number of vessel segments.
3. Vessel density.
4. Tortuosity.
5. The minimum vessel length.
6. The maximum vessel length.
7. The median vessel length.
8. The mean vessel length.
9. The standard deviation length.
10. The number of bifurcation points.
11. Gray level coefficient of variation.
12. Gradient mean.
13. Gradient coefficient of variation.

In addition, in the study by Gil et al. [23], 5 shape features were extracted considering continuous arterial sections and their corresponding segments delimited by tortuosity as follows:

1. The minimum standard deviation of the segments in length pixels considering all arterial sections. Since each arterial section is composed of continuous segments, it is possible to measure the length of each segment and compute the standard deviation for each section. Therefore, if several arterial sections are present in the image, it is possible to obtain statistical measures over the arterial sections.
2. The maximum standard deviation of segments in length pixels considering all arterial sections.
3. The median standard deviation of the segments in length pixels considering all arterial sections.
4. The average of standard deviations of the segments in length pixels considering all arterial sections.
5. The variance of the standard deviations of the segments in length pixels considering all arterial sections.

In addition, 25 shape features were computed as following:

1. Minimum perimeter. The perimeter of an arterial section is the length of its boundary.
2. Maximum perimeter.
3. Median perimeter.
4. Mean perimeter.
5. Standard deviation of the perimeters.
6. Minimum compactness. It can be computed as follows:
  (8) $$\mathrm{Compactness}=\frac{{\mathrm{Perimeter}}^{2}}{\mathrm{Area}}.$$
7. Maximum compactness.
8. Median compactness.
9. Mean compactness.
10. Standard deviation of compactness.
11. Minimum circularity ratio. It can be computed as follows:
  (9) $$\mathrm{Circularity}\;\mathrm{Ratio}=\frac{4\cdot \pi \cdot \mathrm{Area}}{{\mathrm{Perimeter}}^{2}}.$$
  Similar to previous measures, for images containing several arterial sections, it is possible to compute circularity for each section and obtain statistical measurements.
12. Maximum circularity ratio.
13. Median circularity ratio.
14. Mean circularity ratio.
15. Standard deviations of the circularity ratios.
16. Minimum rectangularity. It can be computed as follows:
  (10) $$\mathrm{Rectangularity}=\frac{\mathrm{Area}\;\mathrm{of}\;\mathrm{Arterial}\;\mathrm{Region}}{\mathrm{Area}\;\mathrm{of}\;\mathrm{Bounding}\;\mathrm{Rectangle}\;\mathrm{of}\;\mathrm{Arterial}\;\mathrm{Region}}.$$
17. Maximum rectangularity.
18. Median rectangularity.
19. Mean rectangularity.
20. Standard deviation of rectangularities.
21. Minimum elongatedness. It can be computed as follows:
  (11) $$\mathrm{Elongatedness}=\frac{l}{w},$$
  where *l* is the arterial section length in pixels, and *w* represents the vessel width in pixels.
22. Maximum elongatedness.
23. Median elongatedness.
24. Mean elongatedness.
25. Standard deviation of elongatedness.

Finally, 6 shape-density features were also extracted.

1. Minimum vessel pixel density of all arterial sections present in the patch.
2. Maximum vessel pixel density.
3. Median vessel pixel density.
4. Mean vessel pixel density.
5. Standard deviation of the vessel pixel densities.
6. Sum of the vessel pixel densities of all arterial sections.

A set with 50 distinct shape-based features was described previously. Since shape feature values are dependent on the applied enhancement method, they were extracted from the 8 responses corresponding to each applied enhancement method in order to extract a set with 400 shape-related features.

After the feature extraction process is concluded, a numeric feature dataset is generated and partitioned randomly into training and testing instances in a balanced manner. The feature selection task is performed on the training dataset, and it is turned into a search process that is conducted by the hybrid-evolutionary algorithm involving the BUMDA and SA metaheuristics.

Since the total number of extracted features is 473, the identification of an optimal feature subset using an exhaustive search process involves a computational cost of O($2^{473}$), which is highly difficult to perform. By involving a single search evolutionary method, the problem can be solved partially. However, due to the high-dimensional complexity of the problem, it is possible to improve the solution achieved by the evolutionary method at each iteration, applying a refined search. This will lead to the use of a hybrid-evolutionary method in which the main goal of feature selection is to identify an optimal subset that improves classification accuracy and reduces model complexity.

The proposed method for the automatic feature selection task is formed by the BUMDA and the SA strategies. The use of BUMDA is adequate because the use of the Boltzmann distribution produces spread populations in comparison with the UMDA and other related techniques [35], which also decreases the risk of falling into local-optima solutions. In addition, since the SA algorithm is a single-solution search method, it is suitable to improve the best solution produced by the BUMDA at each iteration in a refined search step. The combination of these metaheuristics produces a hybrid-evolutionary method focused on the search for the best suitable feature subset by considering the minimization of its size and the maximization of the accuracy classification performance.

Since the feature selection task involves the evaluation of each feature subset produced by the search techniques, a classification model must be trained. By considering that BUMDA is governed by the population size (ps) and the number of generations (ng), and the SA is governed by the number of iterations (ni) computed from the initial, final, and step temperature, the total number of classification models to train is computed as ps×ng×ni. For instance, if ps=100, ng=1000, and ni=1000, the total number of different classifiers to be evaluated is $1\times 10^{8}$. Consequently, a fast-convergent classification method such as the K-nearest neighbor is suitable in this stage. Subsequently, when the feature selection stage is completed, a more complex classification technique, such as the SVM, can be used in a testing stage to improve the classification performance.

**Figure 4:**
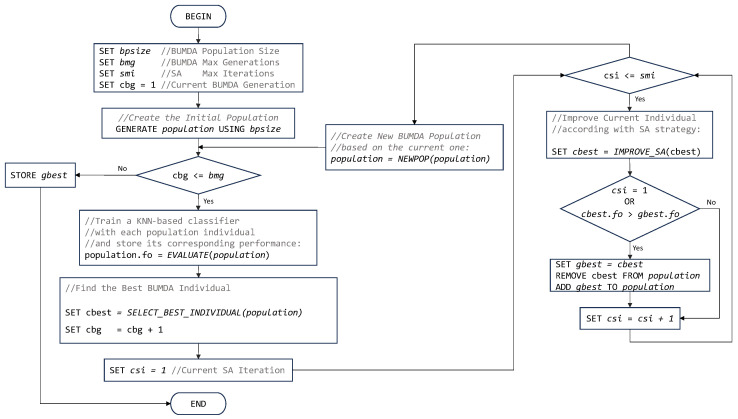
Flowchart of the proposed hybrid method to perform automatic feature selection.

Figure 4 illustrates the flowchart corresponding to the proposed hybrid- evolutionary method.

To evaluate the feature selection performance of the proposed method, the feature decreasing rate (FDR) metric was used and it can be computed as follows:

(12) $$\mathrm{FDR}=1-\frac{\mathrm{Number}\;\mathrm{of}\;\mathrm{Selected}\;\mathrm{Features}}{\mathrm{Total}\;\mathrm{Number}\;\mathrm{of}\;\mathrm{Features}}.$$

The FDR metric is used as a way to measure the feature selection performance. When the number of selected features decreases, the FDR increases, allowing the hybrid-evolutionary method to conduct the search for an optimal feature subset by maximizing the FDR.

**Figure 5:**
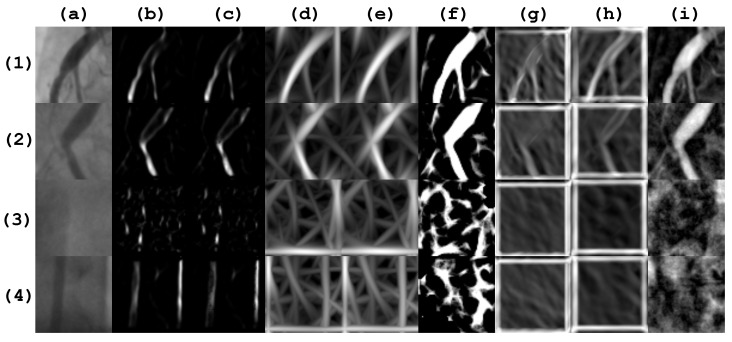
Patch samples from a database of 608 images. Rows (**1**) and (**2**) correspond to positive stenosis cases. Rows (**3**) and (**4**) correspond to negative stenosis cases. Column (**a**) corresponds to the original patch image. Columns (**b**–**i**) correspond to the different vessel enhancement method responses, as follows: Frangi, Salem, simple-scale Gabor, multi-scale Gabor, multi-scale linear, multi-scale matched filter, single-scale matched filter, and top-hat operator.

The final step of the proposed method is the classification of an independent test set using the subset of selected features. In the experiments, the first database of 608 images was divided into training (508 images) and testing (100 images) sets. Both datasets use a balance of positive and negative cases. Each patch is in a grayscale color scheme and their corresponding size is 64×64 pixels. In Figure 5, a subset of sample images, along with its filter response, are presented.

**Figure 6:**
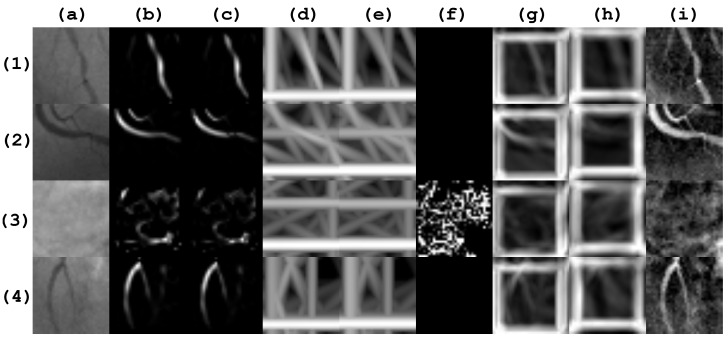
Patch samples from the second database of 2788 images. Rows (**1**) and (**2**) correspond to positive stenosis cases. Rows (**3**) and (**4**) correspond to negative stenosis cases. Column (**a**) corresponds to the original patch image. Columns (**b**–**i**) correspond to the different enhancement method responses as follows: Frangi, Salem, simple-scale Gabor, multi-scale Gabor, multi-scale linear, multi-scale matched filter, single-scale matched filter, and top-hat operator.

In order to evaluate the obtained results, a dataset was formed from the second image database described in Section 2.1. The dataset was divided into the training set with ≈65% (1828 instances) and testing set (960 instances). In Figure 6, sample patches corresponding to the second dataset, are presented.

To determine the classification performance, the accuracy and the Jaccard Coefficient metrics were used. The accuracy metric can be calculated as follows:

(13) $$Acc=\frac{TP+TN}{TP+TN+FP+FN},$$

where Acc is the classification accuracy, TP is the fraction of positive cases classified correctly, TN is the fraction of negative cases classified correctly, FP is the fraction of negative cases classified as positive, and FN represents the fraction of positive cases classified as negative. Moreover, the Jaccard coefficient (JC) is calculated as follows:

(14) $$JC=\frac{TP}{TP+FP+FN},$$

The accuracy and the JC metrics are convenient for measuring binary classification performance. The accuracy metric allows us to know how well the classification of positive and negative stenosis cases is performed. However, in coronary stenosis classification, it is important to know the performance of positive coronary stenosis classification, which is evaluated by the JC metric. These two metrics are the most commonly used in image binary classification problems.

## 4. Results and Discussion

In this section, the results achieved in each stage of the proposed method are presented and discussed. Moreover, they are compared with other methods from the literature. All the experiments were implemented in MATLAB software version 2018 and executed on a computer with an Intel Core i7 processor and 8 GB of RAM.

In the first stage, a bank of 473 features was extracted. This stage involved the original images as well as the responses of the different vessel enhancement methods applied to them. In Table 1, a summary of the distinct feature types extracted is described.

After the feature extraction step was concluded, an optimal subset of features was obtained by applying a hybrid-evolutionary algorithm. The feature subset was evaluated in terms of the training accuracy and FDR metrics. Furthermore, the obtained results were compared with other search methods from the literature. In Table 2, different search methods are described with their corresponding parameter settings, including a statistical analysis of the FDR for each of them.

By considering the results described in Table 2, the highest FDR was obtained using the hybrid-evolutionary strategy, which uses the smallest feature subset of four shape features, as follows:

- Mean Intensity extracted from the Frangi method response.
- Average standard deviation of the segments in length pixels for all arterial sections, extracted from the Frangi filter response.
- Gradient mean extracted from the linear multi-scale method response.
- Gradient coefficient of variation extracted from the top-hat method response.

**Figure 7:**
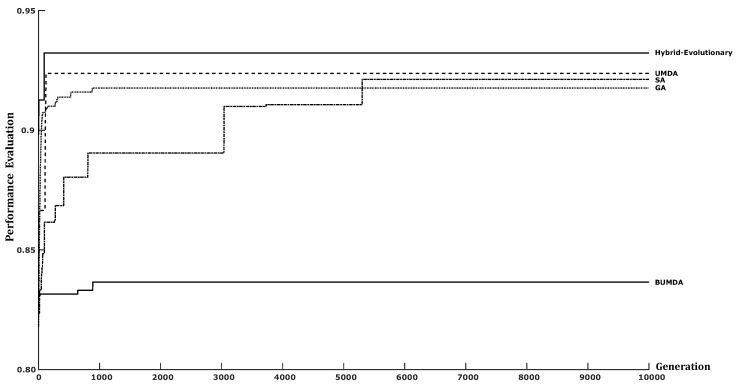
Performance evaluation chart considering the FDR and the training accuracy for selected metaheuristics: UMDA, BUMDA, GA, SA, and the hybrid-evolutionary method.

In addition, the training classification accuracy performance was also considered. For almost all techniques, the FDR variation was small, which means that the search behavior was stable. By involving the FDR and the training classification accuracy, a performance evaluation was conducted. Figure 7 illustrates the performance chart of the best trial for each compared strategy considering the FDR and the training accuracy.

The classification performance of each method was measured using the testing set. In Table 3, the performance obtained using the testing dataset, which corresponds to the first database, is described in terms of the accuracy and Jaccard coefficient metrics. In addition to the KNN-based classifier, an SVM was also trained in order to measure the performance of the selected features under different classifiers. It was established that there was a maximum number of 1000 iterations for KNN and the SVM and a cross-validation with k=10. The values for those parameters were chosen as a tradeoff between an optimal result and the computational time.

The results described in Table 3 shows that the highest rate, in terms of the accuracy and Jaccard coefficient was obtained using the subset of 4 features selected by the hybrid-evolutionary method along with the KNN and the SVM classifiers. Correspondingly, using only 4 of the 473 features represents a discrimination rate of 0.99% over the initial bank of features. Moreover, the obtained results are similar to those achieved using the Deep Learning CNN-16C and GLNet architectures, which exhibits the robustness of the proposed strategy.

To evaluate the efficiency of the previously discussed results, an additional dataset formed from the publically available database [11], was used for training and testing a KNN and an SVM with the bank of 4 features described in Table 4.

**Figure 8:**
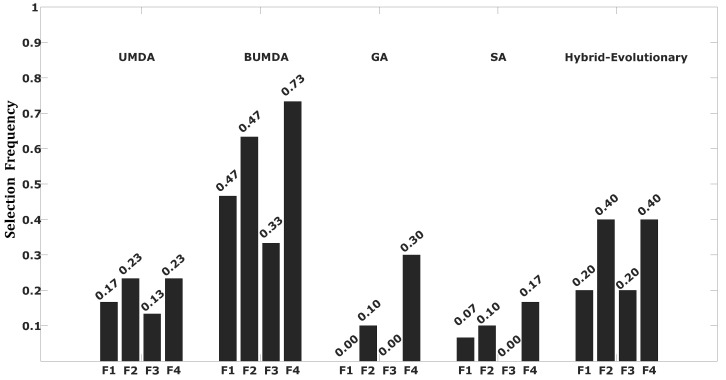
Frequencies for each selected feature in the best solution achieved by the hybrid-evolutionary method contrasted with the frequency for that same feature in the best solution achieved by the other methods. Selected features are as follows: Frangi filter—mean intensity (F1), Frangi filter—mean std. dev. of segments length in all arterial sections (F2), linear multi-scale filter—gradient mean (F3), top-hat—gradient coefficient of variation (F4).

Table 4 shows that the highest classification rate in terms of the accuracy and Jaccard coefficient metrics was also achieved by the proposed method, along with the SVM-based classifier. This result is relevant because it shows that the SVM classification performance is competitive using the bank of four features. Similarly, results obtained using the KNN-based classifier were close to those achieved by the SVM, also using the 4D feature vector. Consequently, the accuracy and Jaccard coefficient rates show how competitive the feature subset was in classifying stenosis cases. An analysis of the frequency for each of the selected features is described in Figure 8.

According to Figure 8, almost all selected features by the hybrid-evolutionary method have a presence in the other contrasted search methods, which provides evidence of their relevance in the classification process.

**Figure 9:**
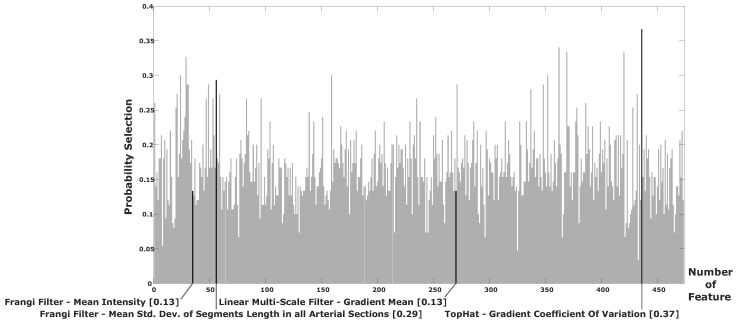
Selection probability for all 473 features based on the frequency in which each of them was selected in all trials considering all contrasted strategies.

Based on Figure 9, it is relevant that two of the four features that were selected by the hybrid-evolutionary strategy are in the group of features with the highest selection probabilities considering all trials of all compared techniques. This finding is important because it statistically validates the relevance of the final selected 4D feature vector for the automatic classification of coronary stenosis cases. In addition, the mean time required for the classification of a single testing instance was ≈0.02 s.

Even when the achieved results were competitive, the weak vessel contrast present in almost all coronary angiograms and the continuous heart movement decreased the response accuracy of vessel enhancement methods, leading to classification errors. Since the human body is composed of a variety of molecular substances, the reaction of the contrast medium to the X-rays is not enough to produce an optimal artery distinction in the majority of the coronary angiograms. However, although technological innovations have been produced to improve the generation of coronary angiograms [47], the elevated cost of medical imaging devices makes it difficult to adopt in the short term.

One of the strengths of the proposed methods is the identification of a subset with relevant features, which allows performing a positive and negative classification of coronary stenosis cases with a high accuracy rate. However, the conformation of the feature bank can be a limitation in the proposed method and also a robust discrete optimization technique for high-dimensional problems.

After the testing stage was finished, the obtained results with the proposed method achieved a high classification performance in terms of the accuracy and the JC metrics using only a 4D feature vector. For instance, using the proposed method with the first testing set, accuracy and JC rates of 0.87 and 0.76 were achieved, respectively. It is relevant that the obtained accuracy was slightly higher than that achieved by the deep learning model proposed by Antczak et al., which was 0.86. In addition, the hybrid-evolutionary strategy results also overcome to those obtained using single search metaheuristics. The single BUMDA and SA methods achieved a classification accuracy performance of 0.82 and 0.85 in their best result. Accordingly, the single SA technique was close to the best accuracy result. However, the single SA found a 15D feature vector with respect to the 4D feature vector found using the hybrid-evolutionary strategy, which shows how the hybrid approach was relevant to producing an optimal feature vector in terms of size and classification performance. Furthermore, when the second testing dataset was used to measure the classification performance, accuracy and JC rates of 0.89 and 0.80 were achieved, respectively, which surpassed those obtained by all the compared methods, including the results obtained with deep learning techniques (0.86 and 0.74 for accuracy and JC metrics) and single metaheuristics.

## 5. Conclusions

In this paper, a novel strategy consisting of three stages was presented for the automatic classification of positive and negative coronary stenosis cases. In the first step, a bank of 473 features was formed, which represents a computational search complexity of O$\left(2^{473}\right)$, which was explored using different search strategies from the literature. Results achieved in this stage were relevant since only 4 of the 473 features (decreasing rate of 0.99) were selected using the hybrid-evolutionary method across all of the other methods compared. It was also relevant that three of the four features are of shape type: Frangi filter—average of the standard deviations of the segments length across all arterial sections, the linear multi-scale filter—gradient mean, and the tophat—gradient coefficient of variation. The remaining feature is intensity type and corresponds to the mean intensity, which is also extracted from the response of the Frangi enhancement method. It is relevant that none of the selected features were obtained from the original image directly. In the testing stage, the proposed method achieved the highest classification performance in terms of accuracy and JC metrics. For the first testing set, the highest accuracy and JC rates were 0.87 and 0.86, respectively. Correspondingly, with the second dataset, the highest performance in terms of accuracy and JC was 0.84 and 0.89, respectively. Additionally, taking into account the computational time and classification accuracy of the proposed method based on four selected features, it can be a potential method as part of a computer-aided diagnosis system in cardiology. Finally, future work can be conducted in fast convergence of feature selection for high-dimensional spaces in order to improve the computational time without decreasing the classification performance rate.

## Figures and Tables

**Table 1:** Summary of the different feature types extracted from the original images and their corresponding vessel-enhancement responses.

Feature Type	Require the Original Image	Require Vessel-Enhancement	Quantity
Intensity	Yes	Yes	45
Texture	Yes	No	28
Shape	Yes	Yes	400
**Total Extracted Features:**			**473**

**Table 2:** Parameter settings and statistical analysis of the feature decreasing rate (FDR) for the feature selection performance of distinct search metaheuristics of 30 independent trials.

Method	Pop. Size	Max. Gens.	Min. FDR	Max. FDR	Median FDR	Mean FDR	Std. Dev. FDR
UMDA	100	1000	0.48	0.98	0.92	0.83	0.17
BUMDA	100	1000	0.57	0.50	0.57	0.53	0.02
GA	100	1000	0.94	0.91	0.94	0.93	0.01
SA ^1^	-	10,000	0.87	0.97	0.92	0.92	0.03
Hybrid Evolutionary ^2^	100	100/5000 ^1^	0.92	0.99	0.97	0.97	0.03

^1^ Simulated annealing $(T_{max}=1,T_{min}=0,T_{step}=0.0001$). ^2^ BUMDA was configured with 100 maximum number of generations. Subsequently, the inner SA strategy was configured with $\left(T_{max}=1,T_{min}=0,T_{step}=0.005\right)$. In total, the hybrid-evolutionary strategy iterated $5\times 10^{5}$ times.

**Table 3:** Description of the test results for each compared strategy using the KNN and the SVM classifiers, including the number of selected features (NSF) with their corresponding feature decreasing rate (FDR), the achieved classification accuracy, and Jaccard coefficient (JC), using the first image database containing 100 balanced instances.

Method	NSF	FDR	Classifier	Accuracy	JC
GLNet [44]	–	–	–	0.85	**0.76**
UNet [45]	–	–	–	0.85	0.75
CNN-16C [11]	–	–	–	0.86	**0.76**
UMDA [46]	10	0.98	KNN	0.81	0.75
			SVM	0.80	0.67
BUMDA	205	0.57	KNN	0.81	0.70
			SVM	0.82	0.72
GA	29	0.94	KNN	0.79	0.65
			SVM	0.79	0.66
SA	16	0.97	KNN	0.81	0.65
			SVM	0.85	0.75
Hybrid-Evolutionary	4	0.99	KNN	**0.87**	**0.76**
			SVM	**0.86**	0.75

**Table 4:** Description of the testing results for each compared strategy using the KNN and the SVM classifiers, including the number of selected features (NSF) with their corresponding feature decreasing rate (FDR), the achieved classification accuracy and Jaccard coefficient, using a dataset formed from the Antczak image database, containing 960 balanced instances.

Method	NSF	FDR	Classifier	Accuracy	JC
GLNet [44]	–	–	–	0.72	0.63
UNet [45]	–	–	–	0.76	0.72
CNN-16C [11]	–	–	–	0.86	0.74
UMDA [46]	10	0.98	KNN	0.85	0.75
			SVM	0.83	0.71
BUMDA	205	0.57	KNN	0.86	0.77
			SVM	0.74	0.56
GA	29	0.94	KNN	0.85	0.75
			SVM	0.87	0.78
SA	16	0.97	KNN	0.85	0.75
			SVM	0.85	0.75
Hybrid Evolutionary	4	0.99	KNN	0.84	0.74
			SVM	**0.89**	**0.80**

## Data Availability

The first database presented in this article are not readily available because ethic and legal restrictions. The second database is not our authorship. It is publicly available at: https://github.com/KarolAntczak/DeepStenosisDetection.

## Abbreviations

The following abbreviations are used in this manuscript:

BUMDA	Boltzmann univariate marginal distribution algorithm
FDR	feature decreasing rate
GA	genetic algorithm
KNN	K-nearest neighbor
NSF	number of selected features
SA	simulated annealing
SVM	support vector machine
UMDA	univariate marginal distribution algorithm
